# Impact of Non-vitamin K Antagonist Oral Anticoagulant Withdrawal on Stroke Outcomes

**DOI:** 10.3389/fneur.2018.01095

**Published:** 2018-12-18

**Authors:** Joong Hyun Park, Sang Won Han, Kyung-Yul Lee, Hye-Yeon Choi, Kyeongyeol Cheon, Han-Jin Cho, Yo Han Jung, Hyung Jong Park, Hyo Suk Nam, Ji Hoe Heo, Hye Sun Lee, Gustavo Saposnik, Young Dae Kim

**Affiliations:** ^1^Department of Neurology, Inje University College of Medicine, Seoul, South Korea; ^2^Department of Neurology, Gangnam Severance Hospital, Severance Institute for Vascular and Metabolic Research, Yonsei University College of Medicine, Seoul, South Korea; ^3^Department of Neurology, Kyung Hee University School of Medicine, Kyung Hee University Hospital, Seoul, South Korea; ^4^Department of Neurology, Pusan National University Hospital, Pusan National University College of Medicine and Biomedical Research Institute, Busan, South Korea; ^5^Department of Neurology, Changwon Fatima Hospital, Changwon, South Korea; ^6^Department of Neurology, Yonsei University College of Medicine, Seoul, South Korea; ^7^Biostatistics Collaboration Unit, Yonsei University College of Medicine, Seoul, South Korea; ^8^Stroke Outcomes and Decision Neuroscience Research Unit, Division of Neurology, St. Michael's Hospital, University of Toronto, Toronto, ON, Canada

**Keywords:** stroke, non-valvular atrial fibrillation, outcome, cardiac embolism, anticoagulation

## Abstract

**Introduction:** Discontinuation of oral anticoagulants such as non-vitamin K antagonist oral anticoagulants (NOACs) may induce a hypercoagulable state, leading to severe stroke and poor outcomes. This study aimed to compare stroke outcomes between NOACs withdrawal and other prior medication statuses in patients with non-valvular atrial fibrillation (NVAF).

**Methods:** Consecutive patients who had pre-existing NVAF and were admitted for an acute ischemic stroke or transient ischemic attack- at five hospitals between January 2013 and December 2016 were included. Prior medication status was categorized into seven groups such as no antithrombotics, antiplatelet-only, warfarin with subtherapeutic intensity, warfarin with therapeutic intensity, NOAC, warfarin withdrawal, and NOAC withdrawal. We compared initial National Institute of Health Stroke Scale (NIHSS) scores between groups

**Results:** Among 719 patients with NVAF, The median NIHSS score at admission was 5 (IQR 1-13). The NOAC withdrawal group had the highest median NIHSS scores at stroke onset [16, interquartile range, IQR (1–17)], followed by the warfarin withdrawal group [11, IQR (1–14, 18)], the no antithrombotic group [5, IQR (1–13, 18, 19)], and the warfarin with subtherapeutic intensity group [5, IQR (1–10, 18, 19)]. A Multivariable analysis demonstrated that NOAC withdrawal was independently associated with higher NIHSS scores at stroke onset (B 4.645, 95% confidence interval 0.384–8.906, *P* = 0.033). The median interval from drug withdrawal to ischemic stroke or TIA was 7 days (IQR 4-15) in the NOAC group.

**Conclusions:** Stroke that occurred after stopping oral anticoagulants, especially NOAC, and was more severe at presentation and associated with poorer outcomes.

## Introduction

Non-valvular atrial fibrillation (NVAF) is the most common etiology of cardioembolic ischemic stroke (20). Although NVAF-related stroke is associated with an increased risk of stroke or fatal stroke, oral anticoagulation may effectively prevent ischemic events including an ischemic stroke or a transient ischemic attack (TIA) (19). Following the publication of studies which showed the benefits of non-vitamin K oral anticoagulants (NOACs) compared to warfarin, NOACs became widely used in clinical practice worldwide (1, 18).

The discontinuation of antithrombotics can be associated with an increased risk of cardiovascular events and poor outcomes (2, 3). Abrupt warfarin withdrawal may induce a rebound or a paradoxical prothrombotic state, subsequently leading to an increased incident risk of stroke for up to 3 months (4). Considering the potent anticoagulant effect of NOACs on the suppression of thrombogenesis, NOAC withdrawal may also be associated with severe stroke at presentation and poor outcomes in NVAF-related stroke. However, to date, there is limited information on the existence of this phenomenon.

The aim of this study was to compare the initial stroke severity associated with NOAC withdrawal to those associated with other prior medication statuses including warfarin withdrawal in patients with NVAF.

## Methods

### Study Population

We retrospectively reviewed the medical records of consecutive patients with an acute ischemic stroke or TIA and NVAF, who were admitted within 7 days at the Department of Neurology at four regional stroke centers (Severance Stroke Center, Gangnam Severance Stroke Center, Kyung Hee University Hospital Stroke Center, and Inje Paik Hospital) and one general hospital (Changwon Fatima Hospital) between January 2013 and December 2016. During hospitalization, all patients underwent brain imaging and were managed through a standardized care pathway based on current guidelines. For each patient, demographic data, previous medical history, vascular risk factors, clinical manifestations, standard blood tests, and underlying vascular disease were systematically investigated.

This study was approved by the institutional review board at each participating hospital, and the requirement for informed consent was waived because the database was accessed only for analysis purposes and personal information was not used.

### Clinical Information

We collected data on demographics and traditional vascular risk factors such as hypertension, diabetes, dyslipidemia, and current smoking status (5). History of previous ischemic heart disease, congestive heart failure, peripheral arterial occlusive disease, or ischemic stroke was also investigated. TIA was defined as a transient episode of neurologic dysfunction caused by focal brain or retinal ischemia without acute infarction on a brain MRI including diffusion-weighted imaging. Data on international normalized ratio (INR) levels, lipid profile at admission, and creatinine clearance rate using the Cockcroft-Gault equation were collected. The blood levels of the NOACs were not measured in this study. We also calculated the CHA2DS2-VASc score in all patients based on their comorbid conditions (6). In patients with acute ischemic lesions on the brain imaging, we determined whether the stroke mechanism was caused by cardioembolism or if it had more than two causes according to the Trial of Org 10172 in Acute Stroke Treatment classification (7).

### Medication Status

Based on medical records or interviews with the patients or their next of kin or caregiver, we determined the medication that was taken prior to the index stroke. Using data on the medications used prior to the stroke and the laboratory results at stroke presentation, we categorized the patients into seven groups as follows: (1) no antithrombotics, (2) antiplatelet-only, (3) warfarin with subtherapeutic intensity (INR < 2), (4) warfarin with therapeutic intensity (INR≥2), (5) NOAC, (6) warfarin withdrawal, and (7) NOAC withdrawal. Warfarin withdrawal and NOAC withdrawal were defined as those patients who had previously taken an oral anticoagulant but had discontinued the drug within 2 months before the index stroke (8). If a patient had stopped taking warfarin or NOACs, we determined the reason for the discontinuation. We also collected data on the prior use of statins before the index stroke.

### Outcome Measures

We collected data on the initial stroke severity at stroke presentation. The neurological status of each patient during hospitalization was determined regularly at our study centers using the National Institute of Health Stroke Scale (NIHSS) scores which were obtained by a senior neurology resident or stroke specialist.

### Statistical Analyses

Statistical analysis was performed using the Windows SPSS package (version 23.0, IBM Corp., Armonk, NY, USA) and the R package (version 3.1.0, http://www.R-project.org). Categorical variables between groups were compared using the chi-square test or Fisher's exact test, while the independent *t*-test or Kruskall-Wallis test was used for the comparison of continuous variables. When we investigated whether NIHSS scores differed between the groups, Tobit analysis was used to determine the factors influencing initial stroke severity. Tobit regression was used to address the ceiling and floor effects noted in clinical outcomes (9). Using Tobit regression analysis adjusted for the potential confounders (*P* < 0.05 on univariable analysis), the independent association between prior antithrombotics and stroke severity was evaluated. Results were expressed as B (95% confidence intervals [CIs]) with the no antithrombotics group as the reference group. Finally, statistical significance was set at *P* < 0.05.

## Results

### Baseline Characteristics

Of the 1,361 patients with atrial fibrillation who were admitted at the study hospitals during the study period, we excluded the patients with valvular heart disease (*n* = 66) and those with atrial fibrillation detected for the first time at hospitalization (*n* = 576). Finally, 719 patients with pre-existing NVAF (707 ischemic stroke and 12 TIA) were included in this study.

Mean age was 73.9 ±10.2 years, and 397 (55.2%) patients were male. Median CHA2DS2-VASc score was 4 (interquartile range [IQR] 3-5). There were 16 patients in the NOAC withdrawal group, 47 in the warfarin withdrawal group, 57 in the NOAC group, 31 in the warfarin with therapeutic intensity group, 130 in the warfarin with subtherapeutic intensity group, 298 in the antiplatelet-only group, and 140 in the no antithrombotic group. Differences in baseline characteristics are summarized in Table 1. The NOAC withdrawal group was more likely to have hypertension and higher CHA2DS2-VASc scores, while the NOAC group was more likely to have a history of ischemic heart disease or ischemic stroke and prior statin use (all P < 0.05).

**Table 1:** Baseline characteristics of study population according to prior medication status.

	**No antithrombotics (*n =* 140)**	**Antiplatelet-only (*n =* 298)**	**Warfarin with subtherapeutic intensity (*n =* 130)**	**Warfarin with therapeutic intensity (*n =* 31)**	**NOAC (*n =* 57)**	**Warfarin withdrawal (*n =* 47)**	**NOAC withdrawal (*n =* 16)**	***P***
Age	72.8 ± 12.1	74.3 ± 10.2	74.1 ± 9.3	75.9 ± 7.8	74 ± 8.7	71.9 ± 10.1	76.5 ± 9.0	0.418
Male	84 (60.0)	166 (55.7)	68 (52.3)	21 (67.7)	24 (42.1)	25(53.2)	9 (56.2)	0.254
Congestive heart failure	25 (17.9)	42 (14.1)	17 (13.1)	8 (25.8)	3 (5.3)	10 (21.3)	3 (18.8)	0.084
Hypertension	98 (70.0)	239 (80.2)	106 (81.5)	29 (93.5)	51 (89.5)	37 (78.7)	15 (93.8)	0.007
Diabetes	39 (27.9)	84 (28.2)	41 (31.5)	5 (16.1)	24 (42.1)	15 (31.9)	3 (18.8)	0.205
Dyslipidemia	27 (19.3)	74 (24.8)	22 (16.9)	11 (35.5)	12 (21.1)	8 (17.0)	6 (37.5)	0.12
Current smoking status	16 (11.4)	31 (10.4)	20 (15.4)	3 (9.7)	5 (8.8)	4 (8.5)	1 (6.2)	0.809
Previous ischemic heart disease	20 (14.3)	90 (30.2)	36 (27.7)	8 (25.8)	21 (36.8)	13 (27.7)	6 (37.5)	0.01
Peripheral arterial occlusive diseases	5 (3.6)	16 (5.4)	8 (6.2)	3 (9.7)	8 (14.0)	3 (6.4)	0 (0.0)	0.159
Previous ischemic stroke	28 (20.0)	70 (23.5)	59 (45.4)	17 (54.8)	39 (68.4)	22 (46.8)	6 (37.5)	< 0.001
Prior statin use	17 (12.3)	118 (39.6)	60 (46.2)	15 (48.4)	34 (59.6)	26 (55.3)	8 (50.0)	< 0.001
Cardioembolic stroke mechanism	120 (86.3)	248 (84.6)	105 (84.0)	2 (71.0)	50 (89.3)	36 (76.6)	15 (93.8)	0.181
Median CHA2DS2-VASc score, median (IQR)	4 (2–5)	4 (3–5)	5 (3–6)	5 (4–6)	5 (4–7)	5 (3–5)	5 (3–5)	< 0.001
CHA2DS2-VASc score								0.001
0	9 (6.4)	6 (2.0)	0 (0.0)	0 (0.0)	0 (0.0)	3 (6.4)	0 (0.0)	
1	17 (12.1)	25 (8.4)	6 (4.6)	0 (0.0)	1 (1.8)	2 (4.3)	0 (0.0)	
≥2	114 (81.4)	267 (89.6)	124 (95.4)	31 (100.0)	56 (98.2)	42 (89.4)	16 (100.0)	
Laboratory findings								
INR	1 ± 0.2	1 ± 0.1	1.4 ± 0.3	2.8 ± 0.8	1.2 ± 0.2	1.2 ± 0.5	1.1 ± 0.2	< 0.001
Creatinine clearance rate	61 ± 26.1	59.7 ± 26.9	60.3 ± 24	55.5 ± 25.2	62.2 ± 21.5	55.2 ± 24	60.2 ± 22.3	0.769
Total cholesterol, mmol/L	4.4 ± 1.0	4.0 ± 1.0	4.0 ± 1.0	3.9 ± 1.0	3.9 ± 1.2	3.7 ± 1.0	3.8 ± 1.0	< 0.001
Triglyceride, mmol/L	1.2 ± 1.0	1.1 ± 0.7	1.1 ± 0.6	1.1 ± 0.4	1.2 ± 0.9	1.2 ± 0.6	0.9 ± 0.3	0.646
High-density lipoprotein, mmol/L	1.2 ± 0.4	1.1 ± 0.3	1.2 ± 0.3	1.1 ± 0.3	1.1 ± 0.3	1.1 ± 0.3	1.2 ± 0.3	0.176
Low-density lipoprotein, mmol/L	2.6 ± 0.7	2.4 ± 0.9	2.3 ± 0.9	2.4 ± 0.9	2.3 ± 1.0	2.1 ± 0.8	2.2 ± 1.0	0.006

*Data are presented as mean (standard deviation) or number (percentages)*.

*NOAC, non-vitamin K oral anticoagulant; IQR, interquartile range; INR, international normalized ratio; NIHSS, National Institute of Health Stroke Scale*.

### NOAC or Warfarin Withdrawal Group

**Figure 1:**
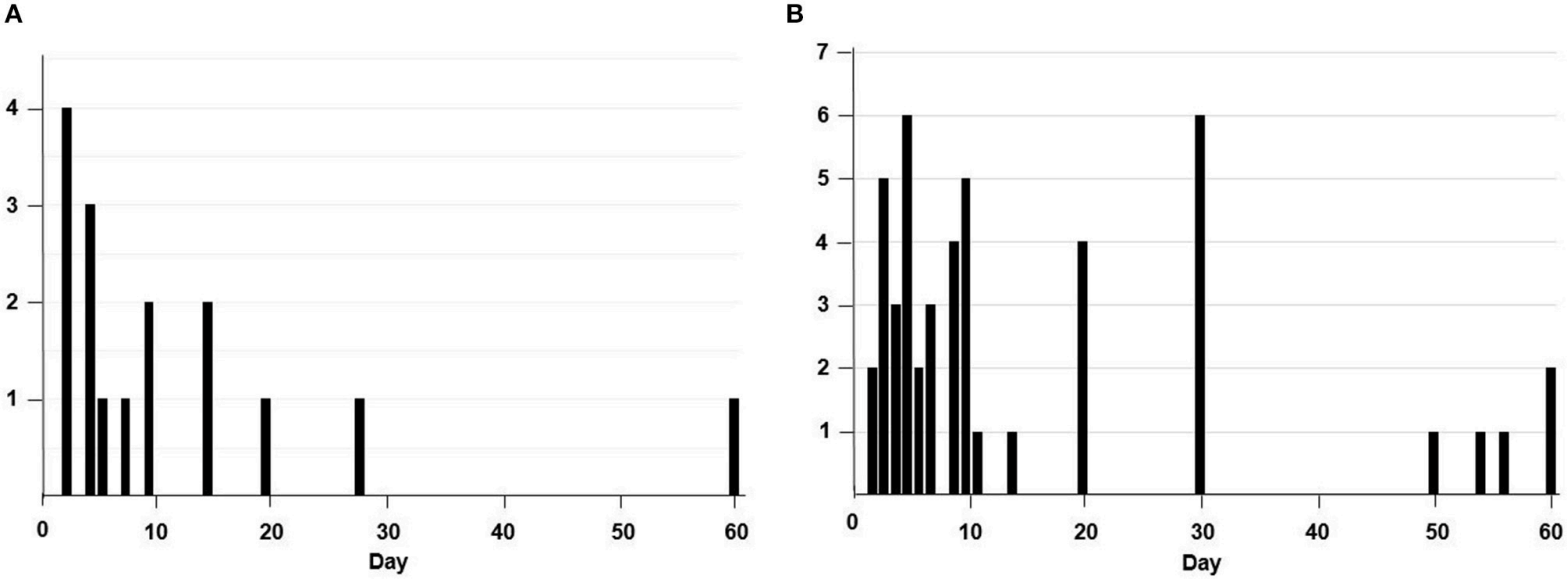
The number of patients who stopped taking non-vitamin K antagonist oral anticoagulans **(A)** or warfarin **(B)** according to the duration (days) of the discontinuation.

The median interval from drug withdrawal to ischemic stroke or TIA was 7 days (IQR 4-15) in the NOAC group and 9 days (IQR 5-20) in the warfarin group (Figure 1). Among the 16 patients who had a stroke after NOAC withdrawal, it occurred in 6 patients after dabigatran discontinuation, 3 after apixaban discontinuation, and 7 after rivaroxaban discontinuation. Among the reasons for NOAC or warfarin cessation, recent bleeding event or self-discontinuation of drug without any specific medical reason was common (Table 2).

**Table 2:** Reasons why oral anticoagulant stopped.

	**NOAC withdrawal (*n =* 16)**	**Warfarin withdrawal (*n =* 47)**
Self-discontinuation without any medical reason	5 (31.3)	28 (59.6)
Recent bleeding event	6 (37.5)	8 (17.0)
Dental procedure	3 (18.8)	3 (6.4)
Endoscopy	0 (0)	5 (10.6)
Scheduled biopsy	1 (6.3)	2 (4.3)
Surgery	1 (6.3)	1 (2.1)

*Data are presented as number (percentages)*.

### Initial Stroke Severity According to the Prior Medication Status

**Figure 2:**
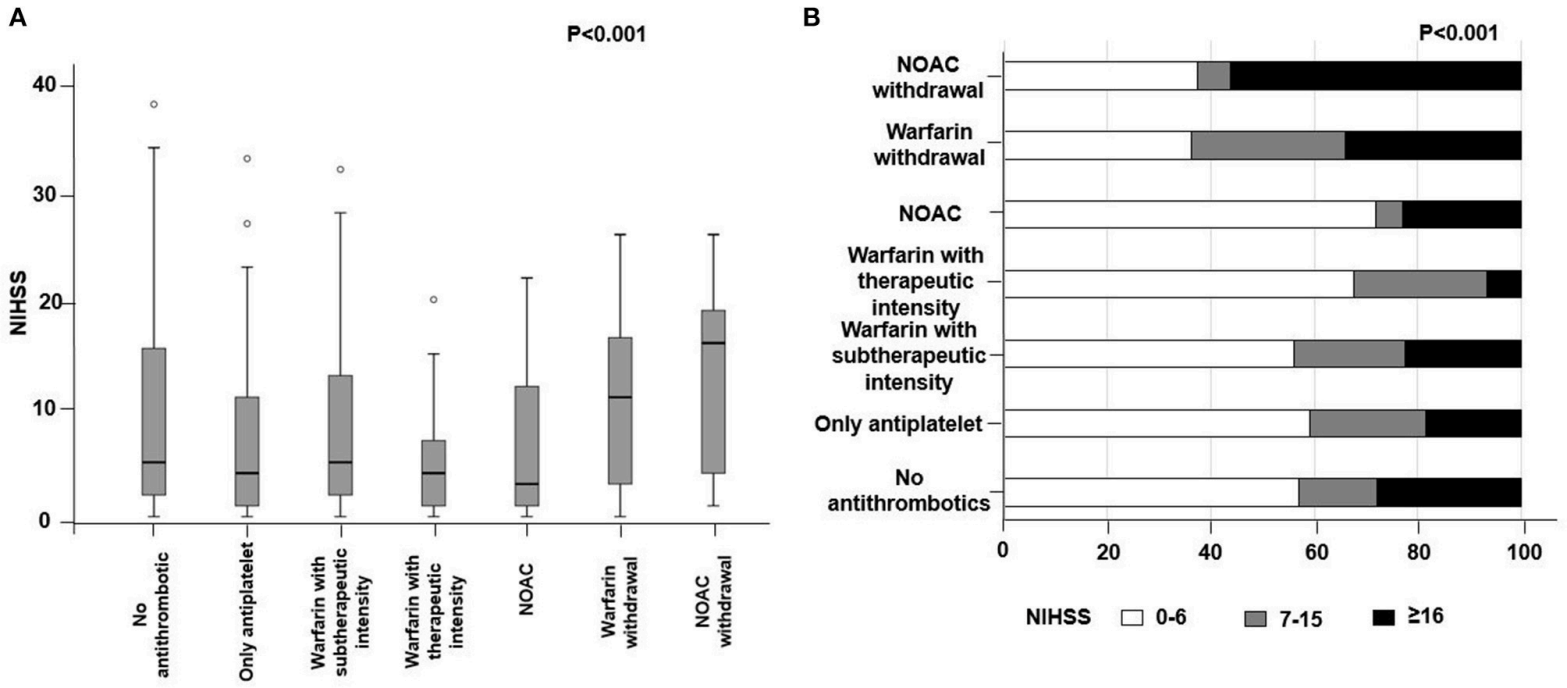
Difference in **(A)** initial National Institute of Health Stroke Scale (NIHSS) and **(B)** stroke severity according to the medication status. NOAC, non-vitamin K antagonist oral anticoagulant.

The median NIHSS score at admission was 5 (IQR 1-13). The NOAC withdrawal group had the highest median NIHSS score [16, IQR (1–17)], followed by the warfarin withdrawal group [11, IQR (1–14, 18)], the no antithrombotic group [5, IQR (1–13, 18, 19)], and the warfarin with subtherapeutic intensity group [5, IQR (1–10, 18, 19)] (Figure 2A and Table 3). When we categorized the stroke severity into three levels such as low (NIHSS ≤ 6), moderate (NIHSS 7-15), and severe (NIHSS ≥16), moderate to severe stroke was more common in NOAC withdrawal or warfarin withdrawal group (Figure 2B). There was no difference in initial NIHSS scores according to the reason for either NOAC withdrawal or warfarin withdrawal.

**Table 3:** Tobit regression analysis showing initial stroke severity according to medication status.

	**Univariable analysis**		Multivariable analysis^*^		**Multivariable analysis**^†^	
	**B (95% CI)**	**P**	**B (95% CI)**	**P**	**B (95% CI)**	**P**
Previous medication						
No antithrombotics	1		1		1	
Antiplatelet-only	−1.584 (−3.283 to 0.115)	0.068	−1.918 (-3.606 to −0.23)	0.026	−1.829 (−3.493 to −0.165)	0.031
Warfarin with subtherapeutic intensity	−0.666 (−2.687 to 1.355)	0.518	−1.021 (−3.042 to 1)	0.322	−0.581 (−2.582 to 1.42)	0.569
Warfarin with therapeutic intensity	−4.359 (−7.693 to −1.025)	0.01	−4.968 (−8.273 to −1.663)	0.003	−4.656 (−7.906 to −1.406)	0.005
NOAC	−1.904 (−4.505 to 0.697)	0.151	−2.365 (−4.958 to 0.228)	0.074	−2.396 (−4.956 to 0.164)	0.066
Warfarin withdrawal	2.844 (0.063 to 5.625)	0.045	2.599 (−0.149 to 5.347)	0.064	2.766 (0.065 to 5.467)	0.045
NOAC withdrawal	5.225 (0.917 to 9.533)	0.017	4.645 (0.384 to 8.906)	0.033	4.297 (0.114 to 8.48)	0.044

*NOAC, non-vitamin K oral anticoagulant*.

* *Adjusted for significant variables in the univariable analysis (P < 0.05) for the entire population (including TIA patients)*.

† *Adjusted for significant variables in the univariable analysis (P < 0.05) for the ischemic stroke patient group*.

The multivariable Tobit regression analysis adjusting for the CHA2DS2-VASc score and current smoking status showed that NOAC withdrawal was independently associated with higher NIHSS scores (B 4.645, 95% CI 0.384–8.906, *P* = 0.033), while prior warfarin with therapeutic intensity (B −4.968, 95% CI −8.273 to −1.663, *P* = 0.003) and antiplatelets only (B −1.918, 95% CI −3.606 to −0.23, *P* = 0.033) were associated with lower NIHSS scores. The results remained consistent when we excluded patients with TIA (*n* = 12) (Table 3) or defined the withdrawal as stopping the drug within 1 month before the index stroke (Supplementary Table 1).

## Discussion

We conducted a large cohort study, compriised of stroke patients with NVAF, to determine the association of NOAC withdrawal and stroke outcomes. Our study revealed that withdrawal of oral anticoagulants, especially NOACs, was associated with higher NIHSS scores at stroke presentation in patients with NVAF. Stroke severity was relatively milder among patients on warfarin with therapeutic intensity or antiplatelets only.

Although there are several studies regarding the risk of thromboembolic events after stopping NOACs (2, 10, 11), the present study is the first to examine stroke characteristics in patients who experienced an ischemic stroke or TIA after abrupt NOAC withdrawal. Previous reports showed that abrupt discontinuation of anticoagulants could cause a rebound phenomenon involving a significant increase in procoagulant markers such as thrombin-antithrombin III complex, fibrinopeptide A and subsequently enhance thrombosis (4, 12, 13). In terms of NOAC withdrawal, some clinical trials, along with several anecdotal reports on patients with deep vein thrombosis or knee replacement surgery, suggested a potential prothrombotic rebound phenomenon after NOAC withdrawal. Most thromboembolic events occurred soon (median 1–2 weeks) following the cessation of dabigatran or rivaroxaban (2, 10, 11, 14, 15). In our study, the median interval between NOAC withdrawal and ischemic events was also 7 days, which may imply the occurrence of a rebound phenomenon associated with NOAC withdrawal.

In our study, stroke severity at presentation was most severe in the NOAC withdrawal group, followed by the warfarin withdrawal group, while prior use of warfarin with therapeutic intensity or antiplatelets alone were associated with lower NIHSS scores. There are some potential explanations to understand this phenomenon. Stroke severity in NVAF is strongly correlated with thrombus characteristics determined by the prothrombotic state in the left atrium and the anticoagulant activity (16, 17). The anticoagulant effect of NOAC decreases rapidly after 12–24 h (21). The transient hypercoagulable state caused by abrupt anticoagulant withdrawal may enhance thrombogenesis, leading to larger sized thrombi. Moreover, thrombin overproduced after cessation of anticoagulation by itself may promote excitotoxic neuronal injury (22, 23). Considering the higher NIHSS scores in the NOAC withdrawal group than in the warfarin withdrawal group, NOAC withdrawal may more strongly suppress thrombin/thrombin activity generation. Together with coagulation activity, thrombus formation in AF can be influenced by other factors including platelet activity (24). The prior use of antiplatelets may prevent thrombus formation in the left atrium and be associated with a milder stroke, although it was not found to be superior to the optimal anticoagulant therapy (25, 26). These assumptions regarding changes in coagulation and platelet activity support our results that demonstrate differences in stroke severity at stroke presentation according to prior medication status.

Although the NOACs have many benefits for clinical use including a fixed dose without the need for frequent monitoring, dietary precautions, and bridging therapy, NOAC use is not always well-maintained in actual clinical practice (18, 27, 28). Among patients requiring NOAC withdrawal because of a planned invasive procedure, the duration of the medication interruption seemed to be prolonged in clinical practice (2). In our study cohort, many patients who stopped medication because of a scheduled invasive procedure, did not re-start medication for a prolonged period, as the time between anticoagulant withdrawal and the ischemic event was found to be long (ranged between 5–20 days in patients using NOACs and 3–56 days in patients using warfarin). Furthermore, patients often stopped anticoagulants without any medical reason. Current guidelines indicated that it is not necessary to discontinue anticoagulant treatment during a low bleeding risk procedure such as dental procedures or diagnostic endoscopy (29). In addition, 18% (3/18) in the NOAC withdrawal group and 17% (8/47) in the warfarin withdrawal group discontinued in these situations. In our study population, 31% in the NOAC withdrawal group and 60% in the warfarin withdrawal group had not specific a medical reason. To improve adherence to medication, the implementation of educational programs and tools to help identify the patients who are more likely to be non-compliant is necessary (18).

Our study had several limitations. First, although we collected data on prior medication status for each patient, there is a possibility that some stroke patients were missed after stopping medication owing to patients' neurologic deficits, especially language dysfunction. However, it was routine practice in our study hospitals for physician or nurses to inquire about the prior medication status at the time of admission with their next of kin or caregiver as well as with the patients themselves. Second, the number of patients included the NOAC withdrawal group was not large in this retrospective study, which could lead to selection bias. The number of TIA patients was also small. In this study, TIA was diagnosed based on a tissue-based definition, not a time-based definition. Previous studies demonstrated that the acute ischemic lesion was detected in over 50% of TIA patients (30). Nearly all patients underwent the brain magnetic resonance image including DWI in this study. Further, although it was not difficult for TIA patients to be admitted to the hospital in Korea, there was still a possibility that TIA patients might be managed on the outpatient clinic, rather than being hospitalized (31). This might have led to the small number of TIA patients in our study population. Third, we did not thoroughly investigate the reason why oral anticoagulation was not resumed. Fourth, this study enrolled only Korean patients, which limited the generalizability of the study findings to other geographic regions or ethnic groups.

Our findings demonstrated that stroke occurred after discontinuing oral anticoagulant medication, especially NOACs, which was independently associated with severe stroke at presentation. Although NOACs offer many advantages for long-term use in patients with NVAF, concerns of adhering to or persisting with NOACs are warranted and physicians should avoid discontinuation of NOACs for prolonged periods (more than 7 days). The reason for NOAC and warfarin withdrawal was unknown in 3 to 6 out of 10 patients with NVAF. Our results revealed the potential harm of NOAC withdrawal which may be useful for physicians to better educate patients and improve the quality of anticoagulation therapy such that effective prevention of thromboembolic events can be achieved in patients with NVAF.

## Author Contributions

JP acquired, analyzed, and interpreted the data, and wrote the original manuscript draft. SH, K-YL, H-YC, KC, H-JC, YJ, and HP acquired and interpreted the data. HN, JH, and GS interpreted the data and provided a critical revision of the manuscript for intellectual content. HL analyzed and interpreted the data. YK conceptualized and designed the study, analyzed and interpreted the data, and provided critical revision of the manuscript for intellectual content.

### Conflict of Interest Statement

The authors declare that the research was conducted in the absence of any commercial or financial relationships that could be construed as a potential conflict of interest.
