# Supplementary material for: Impact of Non-vitamin K Antagonist Oral Anticoagulant Withdrawal on Stroke Outcomes
Source: Front Neurol. 2018 Dec 18;9:1095. doi: 10.3389/fneur.2018.01095 (PMC6305496; doi:10.3389/fneur.2018.01095)
Supplement: Supplementary file 1 [file Table_1.docx]

**Supplementary materials (online only)**

Impact of non-vitamin K antagonist oral anticoagulant withdrawal on stroke outcomes

**Supplementary Table 1.** Tobit regression analysis showing initial stroke severity according to medication status

|  | Univariable analysis | | Multivariable analysis^*^ | | Multivariable analysis^†^ | |
| --- | --- | --- | --- | --- | --- | --- |
|  | B (95% CI) | P | B (95% CI) | B (95% CI) | P | B (95% CI) |
| Previous medication | (0 - 0) |  |  |  |  |  |
| No antithrombotics | (0 - 0) |  |  |  |  |  |
| Antiplatelet-only | -1.5 (-3.178 - 0.178) | 0.080 | -1.781 (-3.449 - -0.113) | 0.036 | -1.702 (-3.344 - -0.06) | 0.042 |
| Warfarin with subtherapeutic intensity | -0.579 (-2.582 - 1.424) | 0.571 | -0.884 (-2.891 - 1.123) | 0.388 | -0.451 (-2.436 - 1.534) | 0.656 |
| Warfarin with therapeutic intensity | -4.271 (-7.591 - -0.951) | 0.012 | -4.830 (-8.123 - -1.537) | 0.004 | -4.523 (-7.759 - -1.287) | 0.006 |
| NOAC | -1.817 (-4.402 - 0.768) | 0.168 | -2.226 (-4.805 - 0.353) | 0.091 | -2.264 (-4.81 - 0.282) | 0.081 |
| Warfarin withdrawal | 3.520 (0.639 - 6.401) | 0.017 | 3.239 (0.391 - 6.087) | 0.026 | 3.391 (0.592 - 6.19) | 0.018 |
| NOAC withdrawal | 5.968 (1.548 - 10.388) | 0.088 | 5.493 (1.118 - 9.868) | 0.014 | 5.151 (0.859 - 9.443) | 0.019 |

NOAC, non-vitamin K oral anticoagulant.

^*^Adjusted for significant variables in the univariable analysis (P<0.05) for the entire population (including TIA patients).

^†^Adjusted for significant variables in the univariable analysis (P<0.05) for the ischemic stroke patient group.
